# Gender Differences in Perception and Care-seeking for Illness of Newborns in Rural Uttar Pradesh, India

**DOI:** 10.3329/jhpn.v27i1.3318

**Published:** 2009-02

**Authors:** Jeffrey R. Willis, Vishwajeet Kumar, Saroj Mohanty, Pramod Singh, Vivek Singh, Abdullah H. Baqui, Shally Awasthi, J.V. Singh, Mathuram Santosham, Gary L. Darmstadt

**Affiliations:** ^1^ International Center for Advancing Neonatal Health, Department of International Health, Bloomberg School of Public Health, Johns Hopkins University, Baltimore, MD 21205, USA; ^2^ Institute of Clinical Epidemiology, King George Medical University, Lucknow, India; ^3^ Department of Pediatrics, King George Medical University, Lucknow, India

**Keywords:** Healthcare-seeking behaviour, Equity, Gender, Health expenditure, Healthcare-use, Neonatal health, Perceptions, Rural health, India

## Abstract

Although gender-based health disparities are prevalent in India, very little data are available on care-seeking patterns for newborns. In total, 255 mothers were prospectively interviewed about their perceptions and action surrounding the health of their newborns in rural Uttar Pradesh, India. Perception of illness was significantly lower in incidence (adjusted odds ratio=0.56, 95% confidence interval 0.33-0.94) among households with female versus male newborns. While the overall use of healthcare providers was similar across gender, the average expenditure for healthcare during the neonatal period was nearly four-fold higher in households with males (Rs 243.3±537.2) compared to females (Rs 65.7±100.7) (p=0.07). Households with female newborns used cheaper public care providers whereas those with males preferred to use private unqualified providers perceived to deliver more satisfactory care. These results suggest that, during the neonatal period, care-seeking for girls is neglected compared to boys, laying a foundation for programmes and further research to address gender differences in neonatal health in India.

## INTRODUCTION

Households and communities in India often place women in subordinate positions to men (1-4). In healthcare, girls are frequently neglected during the care-seeking process, and they experience relatively poorer nutrition, greater delays in receiving care, and lower access to preventative and curative care (3,5-7). A major reason cited for the low girl-to-boy ratio in the population of India (0.93) is the differential in healthcare-seeking behaviour between genders (3,8). Consequently, to improve the status of women and girls in India, it is important to develop interventions that reduce the gender differences in care-seeking, especially during times, such as the neonatal period, when the human body is particularly susceptible to illness and consequences of late or inappropriate health services (9-11). Yet, the organization of such interventions has been limited due, in part, to a lack of documentation on how gender differences express themselves in the care-seeking process during the neonatal period. Moreover, such data are scarce for areas, such as Uttar Pradesh, one of the poorer states of India, where the girl-to-boy ratio (0.90) is one of the world's lowest and where the neonatal mortality rate (53.0 deaths per 1,000 livebirths) is about 120% that of India's average (8,10). The importance of the neonatal period in child health is further reflected by the fact that, of all deaths that occur globally before the fifth birthday, nearly 40% take place during the neonatal period (12).

While existing literature on care-seeking for newborn infants in India has described the female disadvantage in healthcare use-rates, there is a lack of quantitative consideration of potential gender disparities in household recognition of illness, type of care used, and monetary expenditure during the use of health services (7,10). Hospital-based studies have documented that, for every two sick male newborn infants using hospital care, there may be only one sick female counterpart brought for care (10). Community-based studies have also found a significant gender differential in healthcare-use. One study in rural India found that the proportion of sick female and male newborn infants receiving any treatment was 28.8% and 63.1% respectively (7). However, such documentation is not sufficient because gender-based differences could have happened during the recognition of illnesses prior to the use of healthcare. In addition, even when households used healthcare resources for both male and female newborn infants, there may be differences in the quality of care given to each gender. Without such detailed accounts of gender disparities at various levels during the care-seeking process, policy-makers and programme managers could be limited in designing more effective gender-sensitive interventions.

Within this context, the primary objective of this study was to quantitatively assess the gender differences in the perception of any neonatal illness in rural Uttar Pradesh, India. Additional objectives were to quantitatively assess the gender differences in the type and amount of curative care used for sick neonates; describe the role of various household members in the curative care-seeking process; and document the reasons for non-use of curative care for neonates who were perceived sick in rural Uttar Pradesh, India.

## MATERIALS AND METHODS

### Study site

The study was nested in a cluster-randomized trial of the impact of a package of essential newborn care in Shivgarh, a rural block of Uttar Pradesh, India, with a population of 104,000. Households in this area face a pluralist healthcare system consisting of multiple levels of formal care, e.g. primary health centre, community health centre, district hospital, and various allopathy-oriented and private healthcare providers of indigenous characteristics.

### Study participants

Women in the area who became pregnant during the study period were tracked under the demographic surveillance system of the cluster-randomized trial. The parent trial enrolled all expectant mothers who were usual residents of the Shivgarh community, i.e. who had resided in the study area for ≥15 days in succession during the six months prior to delivery, and provided informed consent. Mothers who planned to deliver in a clinic or hospital were excluded from the care-seeking study unless they reached their usual residence within six hours of delivery. In total, 802 households met the eligibility requirements. Of these eligible households, this study focused on 255 of them that were in the comparison arm (i.e. usual care) of the cluster-randomized trial.

The Committee on Human Research at the Johns Hopkins Bloomberg School of Public Health, Baltimore, Maryland, USA, and the Ethical Review Committee at King George Medical University in Lucknow, India, approved the protocol. The trial was registered at: clinicaltrials.gov (#H.22.02.07.15.A1).

### Data collection

Data on care-seeking for newborn's health were collected during February-August 2005. Trained data collectors administered a structured questionnaire in Hindi at the end of the neonatal period, i.e. 28 completed days after birth, for each eligible household that delivered a liveborn neonate during this period. Information about the perception of newborn's illness, the time at which each perceived illness was reported, and associated healthcare-use practices was obtained from the mother of the neonate and was based on a four-week recall period. If care was sought from a healthcare provider, data on the type of care provider, the primary household decision-maker, and expenditure for care-seeking with healthcare providers were recorded. Healthcare providers were defined as individuals who were trained in a particular aspect of healthcare and acted on behalf of households to care for newborns. In the study area, the main types of healthcare care providers were public care providers (e.g. qualified MBBS doctors), private qualified (e.g. MBBS) care providers, *Joala-Chap* providers (i.e. allopathy-oriented healthcare providers who do not have a government-issued medical license), traditional healers, *vaidyas, hakims*, and herbalists. The latter four types of providers were categorized as alternative healthcare providers; *vaidyas* and *hakims* offer services based on the Ayurvedic and Unani traditions respectively. *Joala-Chap* providers were categorized as private unqualified care providers. When payments to healthcare providers for their services were made in kind, the amount of the goods or services was assessed and converted to a monetary value using prevailing market values. When households did not use any care for ill newborn infants, the study obtained information on primary reasons for their actions. Additionally, satisfaction levels with the first healthcare provider used were assessed on a three-point Likert-scale, i.e. unsatisfied, neutral, and satisfied. Variables for satisfaction included satisfaction with ‘overall care’, ‘direct interaction with healthcare provider’, ‘time spent on treatment’, ‘explanation of the immediate treatment’, and ‘explanation of follow-up care’. Household- and village-level information was recorded for each mother-newborn pair enrolled in the study. The socioeconomic status of the household was based on a standard of living index (13).

### Data analysis

Data were double-entered in a Microsoft Access database, cleaned, and imported into the Stata software (version 8) (Stata Corp, College Station, Texas, USA) for analysis (14). Standard descriptive analysis was done first, delineating the sociodemographic characteristics of the study population. Subsequently, to assess the gender differences in the recognition of neonatal illnesses, the study focused on two dependent variables. The first dependent variable was the perception of any neonatal illness during the neonatal period. The denominator for this variable was all eligible neonates enrolled in this study. To evaluate the statistical difference of this variable between male and female neonates, the study initially used a χ^2^ analysis and then a multiple logistic regression analysis, controlling for confounding variables at the household and village levels. To control for clustering effects, generalized estimating equations (GEE) was used (15). The second dependent variable was the age of recognition of the initial perceived neonatal illness. Age of recognition was defined as the number of days between birth and the first perception of illness. The denominator for this variable was all households with perceived sick neonates in this study. The study used a two-sample *t*-test, after assessing for equal variance, to assess for differences in this variable between genders. Moreover, the study assessed the differences in reporting specific perceived neonatal illnesses, i.e. perceived diarrhoea, perceived high fever, etc., using a χ^2^ analysis or Fisher's Exact test, depending on the sample size (16).

In assessing the differences in the type of care used between male and female neonates, the study initially focused on the use of ‘any healthcare resource’ among neonates who were ever perceived ill during the neonatal period. ‘Any healthcare resource’ was defined as the use of either a home-remedy or a healthcare provider. Home-remedies were defined as goods that were fed or applied to a perceived sick neonate. Subsequently, the study focused on all households with neonates ever perceived ill and used a χ^2^ test to assess if there was an overall difference in the form of care sought, i.e. use of home-remedy only, healthcare provider only, healthcare provider and home-remedy combined, or nothing, for perceived sick neonates between genders. Moreover, χ^2^ analysis was used for assessing whether the first type of healthcare provider used differed between male and female neonates. The sample size was insufficient to compare the differences in the use of healthcare provider at the individual illness level.

In the next step of the analysis, the study quantitatively described perceived satisfaction levels of clients and perceived health improvements resulting from the initial use of healthcare provider. We did not analyze satisfaction with healthcare provider by gender due to the small sample size. In measuring the satisfaction levels, a household was considered to be satisfied with a domain of health service if they responded ‘satisfied’ on the Likert scale. A household was considered to be ‘not satisfied’ with a domain of health service if they noted ‘neutral’, or ‘unsatisfied’ on the Likert scale. The differences in healthcare expenditure for the use of healthcare providers across the study arms were also tested using a two-sample *t*-test, after assessing for equal variance. The denominator for the second-step analyses was all perceived sick neonates using a healthcare provider.

Further, the study described whether gender of the neonate had any effect on the role of household members in neonatal care through identifying the primary household decision-maker in situations where the newborn infant was perceived to be sick. A χ^2^ analysis was conducted to ascertain any differences in the role of family members in decision-making for neonatal health by gender. The study concluded with a description of gender differences around reasons for non-use of curative care among households not seeking care for their perceived sick neonates.

## RESULTS

### Subjects

The table delineates the characteristics of the study population. In general, close to one-half of the mothers (n=114) were not literate, three-fourths of the households came from a low socioeconomic background, and approximately nine in 10 households were from the Hindu religious background.

### Perceived neonatal morbidity

The majority (62%) of the neonates (n=158) in the study were perceived ill by their mothers during the neonatal period. The overall rate of perceived illness was significantly lower among households with female (56%, n=70) than those with male neonates (68%, n=88) (p=0.05). The rate of perceived multiple morbidity also tended to be lower among households with female (26%) compared to those with male newborn infants (35%) (p=0.12).

**Fig. 1.**
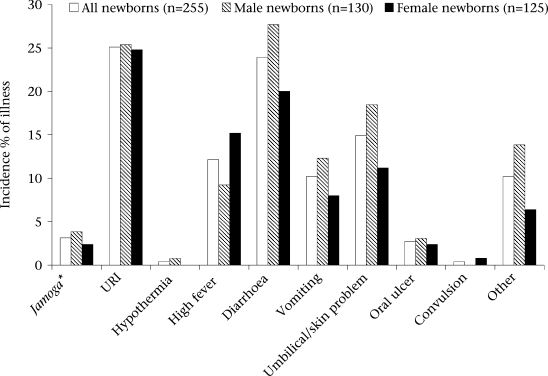
Incidence of perceived neonatal morbidities during the neonatal period by gender

On average, recognition of the first illness of a newborn occurred later among households with females (11.3±8.2 days after birth) than those with males (9.4±7.8 days) (p=0.06). The rates of perceived diarrhoea (p=0.15) and umbilical or skin problems (p=0.10) tended to be lower among households with female neonates compared to those with males (Fig. 1). In contrast, the rate of perceived high fever tended to be higher among households with females compared to those with males (p=0.11).

∗An indigenous illness often associated with evil spirits; URI=Upper respiratory infection

### Association between gender and perceived neonatal morbidity

After controlling for possible confounders through multiple logistic regression analysis, gender remained significantly associated with perception of newborn illness; female newborns were significantly less likely to be perceived ill compared to male newborns [adjusted odds ratio (OR)=0.57, 95% CI 0.34-0.96] (Table). The perception rates for neonatal illness did not vary significantly by maternal education level or age, socioeconomic status of the family, religion/caste, or household size.

**Table.** Sociodemographic characteristics and multiple logistic regression (MLR) for factors associated with perceived neonatal illness

Independent variable	Demographic characteristics (n=255)		Perceived neonatal illness (n=255)		MLR: adjusted odds ratio (95% CI)
	Frequency	%	Frequency	%	
Gender of newborns					
Male	130	51.0	88	67.7	1.00
Female	125	49.0	70	56.0	0.57∗ (0.34-0.96)
Educational status of mothers					
Literate	141	55.3	91	64.5	1.00
Non-literate	114	44.7	67	58.8	0.84 (0.46-1.53)
Socioeconomic status					
Low	196	76.9	120	61.2	1.00
Middle or high	59	23.1	38	64.4	0.94 (0.56-1.93)
Religion/caste					
Hindu/scheduled	99	38.8	60	60.6	1.00
Hindu/non-scheduled	133	52.2	86	64.7	1.30 (0.73-2.33)
Other	23	9.0	12	52.2	0.75 (0.29-1.93)
Age (years) of mothers					
<20	46	18.0	24	52.2	1.00
≥20	209	82.0	134	64.1	1.46 (0.73-2.91)
Household size					
≤4	44	17.2	28	63.6	1.00
5-8	147	57.7	91)	61.9	1.00 (0.41-2.24)
≥9	64	25.1	39	60.9	0.95 (0.41-2.24)
Population of village					
<2,500	149	58.4	99	66.4	1.00
≥2,500	106	41.6	59	55.7	0.64 (0.39-1.06)

^∗p<0.05; CI=Confidence interval; MLR=Multiple logistic regression^

### Use of healthcare

**Fig. 2.**
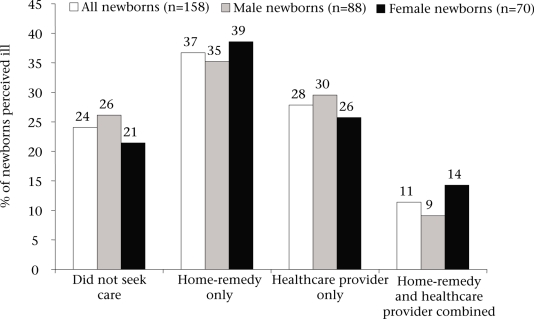
Use of healthcare for newborn infants perceived ill

Overall, 76% of neonates ever perceived ill during the neonatal period received ‘any healthcare resources’. The proportion of male (74%) and female neonates (79%) ever perceived ill and receiving ‘any healthcare resource’ did not differ significantly (p=0.49). Also, there was no gender difference in the general type of care provided to neonates ever perceived ill during the neonatal period (p=0.66) (Fig. 2). The rate of use of a healthcare provider was 40% and 39% for households with sick female and male newborn infants respectively. Thirty-seven percent of all households with perceived ill newborn infants used solely traditional home-remedies while 28% used healthcare providers only, and 11% used a combination of home-remedies and healthcare providers.

p=0.66, X^2^=1.61 (df=3); df=Degree of freedom

At the specific illness level, there was no significant gender difference in the use of healthcare providers (data not shown). Overall, a healthcare provider was sought in the highest proportion of cases of *jamoga* (100%), an indigenous term for a life-threatening illness attributed to ‘evil spirits’, high fever (52%), or a skin/umbilical cord problem (36%).

### Types of healthcare providers used

**Fig. 3.**
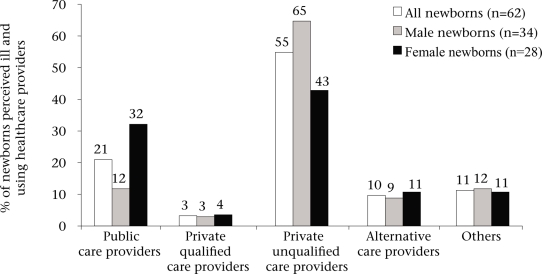
First use of healthcare provider for neonates perceived ill

Overall, 87% of the households using a healthcare provider travelled to see their providers (n=54) while the remaining 13% (n=8) received home-visits. Fifty-five percent of the households using a healthcare provider initially sought a private unqualified provider while 21% initially took their ill newborns to a public-health facility, and only 10% and 3% first consulted a provider of alternative care and a qualified private provider respectively (Fig. 3). There was no overall statistically significant (p=0.34) gender difference in the type of first healthcare provider used. However, there was a trend where the households tended to use private unqualified providers more often for male neonates (65%) compared to female neonates (43%) perceived to be sick (p=0.08). In contrast, public care providers were used more frequently for female (32%) than male neonates (21%) perceived to be sick (p=0.04).

p=0.34, X^2^=5.75 (df=4); df=Degree of freedom

### Characteristics of first healthcare provider

**Fig. 4.**
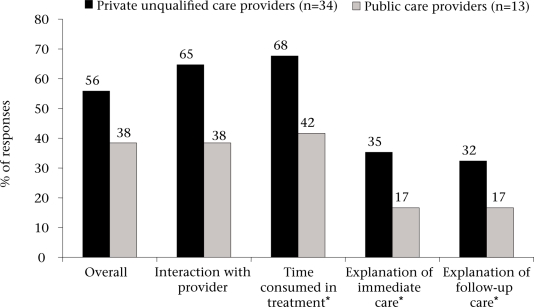
Satisfaction with various aspects of the first realized neonatal health services: private unqualified care providers vs public care providers

Fifty-six percent of the households using private unqualified care providers were satisfied with the ‘overall care’ they received (Fig. 4) while 38% were satisfied with public care providers (p=0.28). Households tended to be more satisfied with the interactions, time spent, and ‘explanations’ of neonatal care by private unqualified providers compared to public care providers, although these differences were not statistically significant. The health of the newborn infant reportedly improved for 85% and 46% of the households initially using private unqualified care providers and public care providers respectively (p=0.01). When all the enrolled mothers were asked what type of healthcare provider they thought was ‘best’ in delivering neonatal health services, 75% cited private unqualified doctors while 16% cited public care providers.

∗One household using a public healthcare provider did not report their satisfaction level for this category

### Healthcare expenditure

The average expenditure in using healthcare providers to treat neonatal illnesses was Rs 163.1 (±411.7) or about US$ 3.50. The average amount spent for male newborn infants, however, was nearly four-fold higher (Rs 243.3±537.2) than for female newborn infants (Rs 65.7±100.7) (p=0.07). The median expenditure in using healthcare providers to treat neonatal illnesses was Rs 22.5 or about US$ 0.50. The median amount spent for male and female newborn infants was Rs 30 and Rs 18 respectively. The average expenditure per consultation with public care providers, private unqualified care providers, and alternative care providers was Rs 3.0 (±5.3), Rs 87.3 (±204.1), and Rs 7.8 (±8.0) respectively. The median fee charged by public care providers, private unqualified care providers, and alternative care providers was Rs 1, Rs 30, and Rs 7.5 respectively. Two households—one with a male neonate and the other with a female neonate—used a private qualified care provider for their ill neonates; one incurred a cost of Rs 200 while the other paid Rs 2,000. The latter cost was associated with a household seeking care for diarrhoea of their newborn infant.

### Household decision-makers in the use of healthcare providers

Fifty-five percent of final decisions to use a healthcare provider were made by a household member other than the mother. The husband and mother-in-law made the final decision to use a healthcare provider in 31% and 18% of the cases respectively. There was no statistically significant difference in the distribution of primary household decision-makers between male and female neonates.

### Reasons for not seeking care

Of households that did not seek any care for a perceived neonatal illness, 80% and 69% with female and male newborn infants respectively primarily cited that they “did not think that care was necessary” (p=0.48). A minority of households with female newborn infants reported that they did not seek care because there was ‘no transportation’ (7%). For male newborn infants, other cited reasons included: ‘God's will' (4%), ‘no transportation' (4%), ‘father was away' (4%), and ‘lack of money' (4%).

## DISCUSSION

In rural Uttar Pradesh of India, mothers were 43% less likely to report illness in female than in male newborn infants, and the time to recognize the initial illness in females was approximately two days longer than in males. The lower likelihood of mothers perceiving illness in female compared to male newborn infants may be because mothers were more vigilant to the health of their sons (17,18). Such gender bias in perception of illness is consistent with findings of other neonatal health studies in South Asia (19). However, it is also possible that the true rate of illness was lower in female neonates. Brothwood *et al*. has documented the relative vulnerability to morbidity of male newborn infants of low birthweight compared to their female counterparts (20). Because the prevalence of low birthweight is relatively high in rural India; the differential rates of perceived illness could be due to true biological differences between the two genders (21). To better understand the reason for the differential rates of perceived illness between male and female newborn infants, future studies should use health professionals to validate the incidence of various morbidities.

There was no significant gender difference in the extent of healthcare used once newborn infants were perceived to be ill. This finding contrasts other reports that found significant differences in healthcare-use between genders (7,10). While this finding is promising for gender equity, it is important to realize that female newborn infants were taken to healthcare providers who were cheaper and perceived to deliver less satisfactory care, and households spent only one-quarter of the monetary resources for female compared to male newborn infants during the healthcare-use process. Yet, such differences may be partially attributed to the relatively-higher rate of multiple morbidities in male neonates. Nevertheless, the notion that households with female newborn infants tended to use public healthcare providers with potentially lower satisfactory care suggests that female newborn infants were more likely to be subjected to lower-quality care. Thus, these results confirm the household's negligence towards girls compared to boys in India, a disparity that begins in the first month of life. In the future, to make more definitive conclusions about this topic, it would be important to objectively measure the quality of care across types of care providers.

Beyond gender-based differences in care-seeking, the majority (62%) of infants were perceived ill at some point during the neonatal period. Upper respiratory infection (URI), diarrhoea, and skin/umbilical problems were the most commonly-cited illnesses. Care was sought in the highest proportion of cases for fever and infections of the skin/umbilical cord. The tendency of families to seek care for problems of the skin and umbilical cord highlights the importance of developing evidence-based clinical algorithms for recognition of cases needing care at a health facility and for treatment regimens appropriate for the community and health facility (22). The reported incidence of URI (25%) was comparable with that from the prospective home-based surveillance using trained village health workers in rural Maharashtra, India, reported by Bang *et al*. (23). However, our reported rate (24%) of diarrhoea was considerably higher than that (5.5%) found by SEARCH while rates of other illnesses from this study, especially hypothermia, were lower (23). The differences in patterns of illness could have arisen from the differences in perception of mothers about signs of newborn illnesses or inherent disparities in the true distribution of disease between the two areas. Given the implications of sensitivity and specificity of recognition of illness on misallocation of healthcare resources, it is important to further evaluate the impact of teaching danger signs to families on ability to recognize illness.

Observations of the overall use-rates of healthcare providers showed a strong preference for non-traditional care providers, such as allopathy-oriented private unqualified care providers, i.e. *Joala-Chap* providers, and alternative care providers, particularly the former. This is consistent with findings of other studies in South Asia (7,11,19,24). A greater use of private unqualified care providers may be because the households were generally more satisfied with their health services, despite their higher consultation fees compared to public care providers. To more rigorously compare the characteristics of neonatal health services among types of providers, it is important that future studies obtain a larger sample size and develop a valid and reliable measurement scale on characteristics of neonatal health services. Through creation of such a scale, decision-makers can identify areas of neonatal health services that are currently not being provided and can devise strategies to increasing the capacity of care providers in these areas, thus promoting sustainable client-provider relationships and better neonatal health outcomes.

It is also important to note that a major reason for not seeking any care for perceived sick neonates was cited as: ‘did not think care was necessary'. Such behaviour is consistent with another report from India, denoting that mothers often did not perceive neonatal illnesses serious enough for use of healthcare provider (10). Consequently, because ill neonates are especially vulnerable to a lack of care, it is important for future studies to qualitatively document why certain households find it unnecessary to seek any care for sick neonates.

A major limitation of this study was that the sample size was relatively small, preventing the detection of significant differences in certain outcomes. The definitiveness of the study results is also limited by the subjective measurements of certain outcomes, i.e. perception of neonatal illness. Yet, given that healthcare-use is often based on subjective behaviour, these results provide important implications for how future interventions can potentially promote greater equity in neonatal health in rural India. The results of the study could also be biased due to recall and misclassification. Since the survey was administered at the end of the neonatal period, the respondents may not have accurately remembered how they perceived illness of their neonate or what actions they took in caring for him/her earlier in the neonatal period. Such recall bias could have led to an inaccurate or underestimation of care-seeking behaviour for newborns. However, as Sudman and Bradburn have found, recall bias was likely minimized through the face-to-face questionnaire format in this study, which aided memories of the respondents (25). Misclassification bias may have occurred because mothers could have misrepresented what actually happened to create a more socially-desirable impression to the interviewers. For instance, mothers could have under-reported the use of alternative care providers due to their reluctance to acknowledge these practices to relatively highly-educated interviewers.

The generalizability of the results may be limited because the study excluded those families that delivered their newborns at health facilities and stayed for over six hours after delivery. Thus, the results of the study may reflect the behaviour of those who are more economically disadvantaged and less apt to use modern healthcare services. However, since about 90% of all births occur at home in rural Uttar Pradesh, the results of the study possess relatively strong generalizability to the study region of Shivgarh (17).

Healthcare-use patterns could also be biased because the survey did not collect information beyond the neonatal period and may have missed care-seeking behaviour for those illnesses that happened towards the end of the neonatal period. Yet, this bias is most likely minimal because the large majority (81%) of newborn illnesses were initially perceived during the first three weeks of the neonatal period. And when an illness happened during the last week of the neonatal period, the study found that it was able to collect care-seeking data from the majority of these households. Further, the results of this study may not reflect behaviours of those mothers who had their babies and subsequently decided to move away from the study site to their parent's home, particularly the maternal parental home. Healthcare-expenditure data may also be biased because the study could not collect reliable and valid indirect healthcare costs. Another important limitation is that this analysis focused on those neonates who survived sex-selective abortion-killing practices and, thus, does not reflect a complete picture of gender discrimination during the neonatal period. Finally, since the study did not cover an entire year, the results of the study did not capture seasonal differences.

In summary, during the neonatal period, households tended to neglect girls compared to boys in the care-seeking process. Neglectful care-seeking behaviour occurred in newborn care as early as the time of recognition of initial illness. The results of this study form a strong foundation for future programmes and for research in understanding gender differences in neonatal health and for the subsequent formation of gender-sensitive health interventions in neonatal health.

## ACKNOWLEDGEMENTS

The authors thank the Shivgarh community for their willing participation. Support was provided by the United States Agency for International Development Mission in India (Delhi) and the Office of Health, Infectious Diseases, and Nutrition, Global Bureau through the Global Research Activity Cooperative Agreement with the Johns Hopkins Bloomberg School of Public Health (No. GHS-A-00-03-00019-00); and the Saving Newborn Lives program of Save the Children-USA through a grant from the Bill & Melinda Gates Foundation. The opinions expressed herein are those of the authors and do not necessarily reflect the views of the US Agency for International Development. The authors also thank Dr. William Reinke, Dr. Kevin Frick, Dr. Saifuddin Ahmed, and Dr. Hugh Waters of the Johns Hopkins Bloomberg School of Public Health and Dr. R. Ahuja, Dr. Mitra, Dr. V.K. Srivastava, and Dr. Narendra Bhandari of King George Medical University for their support and assistance.
